# Blood donation practice and its associated factors among Polish population: secondary data analysis

**DOI:** 10.3389/fpubh.2023.1251828

**Published:** 2023-10-19

**Authors:** Barbara Siekierska, Lucyna Tomaszek, Paulina Kurleto, Edyta Turkanik, Wioletta Mędrzycka-Dąbrowska

**Affiliations:** ^1^Faculty of Medicine and Health Sciences, Andrzej Frycz Modrzewski Krakow University, Kraków, Poland; ^2^Pediatric Division, Institute for Tuberculosis and Lung Diseases, Rabka-Zdrój, Poland; ^3^Department of Anaesthesiology Nursing and Intensive Care, Faculty of Health Sciences, Medical University of Gdańsk, Gdańsk, Poland

**Keywords:** blood donation, donor, determining factors, Polish population, personal value, Satisfaction with Life Scale, blood donors, perioperative

## Abstract

**Introduction:**

Blood transfusion is an intervention widely used in therapeutics (e.g., in the perioperative period), thus, knowledge about factors associated with blood donation is important. The aim of this study was to investigate the impact of sociodemographic factors, personal values and life satisfaction on blood donation in Poland.

**Methods:**

Secondary analysis of data. A multiple logistic regression was carried out to assess the relationship between sociodemographic factors, life satisfaction (Satisfaction with Life Scale), personal values (Personal Values List) and blood donation.

**Results:**

Of the 770 respondents aged 18–65 years, 262 respondents (34%) donated blood at least once in their lives. Respondents who believed that blood donation is safe (OR = 1.71; Cl95%: 1.20 to 2.43), were male (OR = 1.47; Cl95%: 1.26 to 1.72), married (OR = 1.31; Cl95%: 1.11 to 1.54) and those with high school education (OR = 0.81; Cl95%: 0.66 to 0.99) were more often blood donors.

**Conclusion:**

Perceived blood donation safety and sociodemographic factors such as gender, marital status, and education level may influence blood donation. Health education is necessary to increase knowledge and shape positive attitudes toward blood donation among the society.

## Introduction

Blood transfusion is a procedure mainly used in the operating block, perioperative care, as well as in other hospital wards. Among the most common indications for periprocedural transfusion of blood and blood products is anemia associated with blood loss during the procedure, comorbidities or the general condition of the patient before surgery. Anemia, apart from blood loss, usually results from hemoglobinopathies, deficiencies resulting from improper nutrition, but can also be associated with kidney diseases (1). Among the older population, anemia also has its roots in inflammation and blood loss resulting from pathologies of the gastrointestinal tract (2).

The blood and blood products management are determined by each country's law. In Poland, these procedures are regulated by the Act on Public Blood Service of 22nd August 1997 (3). According to this law, certain administrative units may legally obtain blood and prepare blood components: 21 Regional Blood Transfusion Centers, and two centers supervised by the Ministry of Defence and Ministry of Internal Affairs accordingly. They are all a part of the Public Blood Service which is coordinated and organized by the Ministry of Health, National Blood Center and the Institute of Hematology and Transfusion Medicine (IHIT). Additionally, the abovementioned units may use mobile blood collection centers located in specially equipped buses (4).

Legal restrictions for becoming a blood donor in Poland are age (18–65), and meeting the general physical and health conditions assessed by a doctor before donation. Blood donation is a voluntary and unpaid procedure (3)—similarly, free blood donation is also carried out in other European countries, especially those associated in the structures of the European Blood Alliance, which include Belgium, France, Germany, Italy, Sweden and many others (5). In Poland, after donating blood a person becomes an “Honorary Blood Donor,” which entitles them to indirect financial benefits such as: leave from work on the day of the donation and on the next day, reimbursement of travel costs to the Blood Donation and Blood Treatment Center, a regenerative meal and the possibility of tax deduction in a given tax year (tax relief) (3).

According to the World Health Organization (WHO), between 2013 and 2018, the consumption of blood products increased in all regions of the world (6). In addition, in the developed countries (including Poland), the largest group of recipients are people over 60 years of age (6), which in the context of an aging society may indicate a growing trend in the demand for blood and its components.

According to the data of the Polish National Blood Center, in 2021 615,927 people donated blood in Poland, which constitutes about 1.6% of the Polish population (7, 8). This percentage does not ensure self-sufficiency in the supply of blood and its components, which for a European country should be around 2.5% of the population (9). This is why educational social campaigns to encourage potential donors are vital to meeting the basic demands for blood. Such a campaign had begun in 2021 under the name “Your Blood. My life.” This campaign is planned to be implemented by the Polish Government in the years 2021–2026 and is a continuation of previous campaigns. Its goal is to ensure blood and blood components self-sufficiency by the Republic of Poland (10).

Adequate blood supplies are essential for the proper functioning of health systems in the world (11). The maintenance of appropriate blood and blood product resources in health care systems is influenced by many organizational, financial, legislative and safety-related factors (6). Researchers in many regions of the world are trying to determine what factors influence decisions to donate blood both in the group of existing donors and people who are not blood donors (12–14). Attention is paid to, among others, demographic, cultural and religious factors (14, 15). Among the most common factors motivating blood donors for subsequent donations are: knowledge, altruistic factors, the awareness that someone is in need, and the influence of friends and family members who have already donated blood (14–16). The authors of one of the meta-analyses on the motivational factors affecting blood donation suggest that these factors can be divided into eight groups: the convenience of collection site, pro social motivation, personal values, perceived need for donation, indirect reciprocity, marketing communications, incentives, and social norms (15, 17).

The results of a literature systematic review by Piersma et al. regarding individual, contextual and network characteristics of blood donors and non-donors were inconclusive. The collected and analyzed data on donor motivations and demographics vary by country, study and sample characteristics (18), whereas understanding donor profiles is crucial for blood donor management. Therefore, the study was planned, the aim of which was to analyze the factors affecting the state of blood donation in Poland, taking into account socio-demographic factors, personal values and life satisfaction. To the best of our knowledge, the current study was the first in Poland to examine the association between personal values/life satisfaction and being a blood donor.

## Materials and methods

### Study design, setting

**Figure 1:**
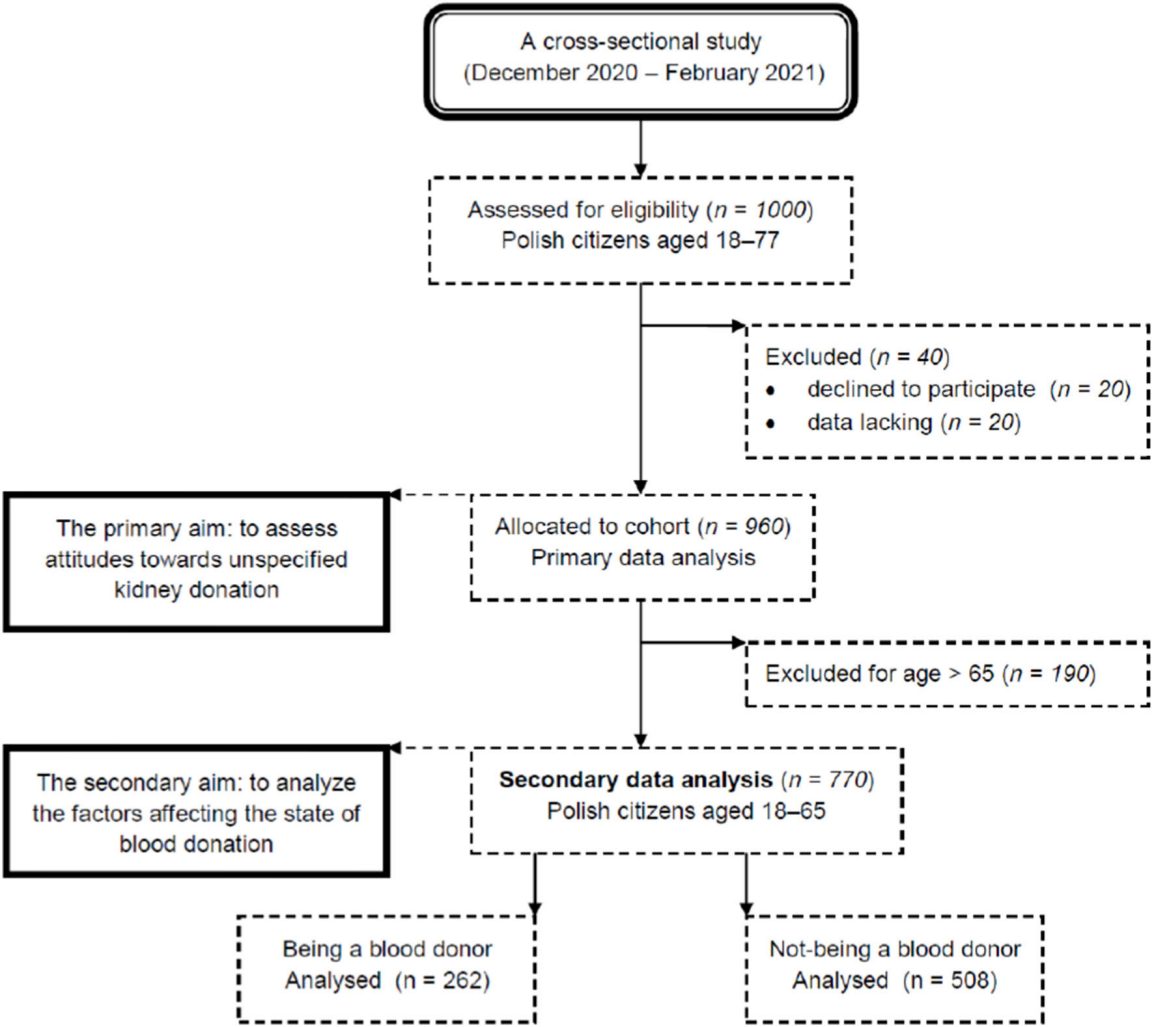
Flow diagram describing secondary data analysis process.

This was a secondary data analysis of a cross-sectional study registered at ClinicalTrials.gov (ID: NCT04789122) with the primary aims to assess attitudes toward unspecified kidney donation among adult Polish citizens. Primary datasets were collected between December 2020 and February 2021 after the approval of the Bioethics Committee of the Andrzej Frycz Modrzewski Krakow University (decision no. KBKA/51/O/2020). The sample size of the mentioned study (*n* = 960) represented the entire Polish population, taking into account age, gender, education and employment status. The secondary data collection included a group of 770 participants aged 18 to 65, because this is the age at which one can become a blood donor in Poland (Figure 1). The total number of participants was then divided into two groups: blood donors (*n* = 262) and non-donors (*n* = 508). The guidelines of the Helsinki Declaration (19) and STROBE (Strengthening the Reporting of Observational Studies in Epidemiology) (20), as well as The General Data Protection Regulation (19) were followed.

### Participants

The studied group included residents of all 16 Polish voivodeships of the ages between 18 and 65 of both sexes. The reasons for exclusion were: age under 18 or over 65, inability to communicate in Polish and lack of Polish citizenship. The respondents were not gratified in any way and gave their voluntary consent to take part in the study.

### Instruments

The study used a diagnostic survey with a questionnaire technique (Computer-Assisted Web Interview). The questionnaires were distributed via the Internet by the Office for Statistical Research and Analysis (Rzeszów, Poland) to fit the criteria of inclusion. Requests to complete the survey were sent to randomly selected Poles inhabiting all 16 Polish voivodships who agreed to receive various offers, including surveys. The database is constantly updated and has been properly prepared for the needs of the research. Also, a self-report questionnaire was used, which included a sociodemographic data sheet and questions about the attitude of the respondents toward blood donation. These questions (#10 Have you ever donated blood in your life? and #11 Do you think blood donation is safe for the donor?) were an integral part of a questionnaire regarding living kidney donation, which is attached in Supplementary Information (21). Standardized tools were the Personal Values List (22) and the Polish version (22, 23) of the Satisfaction With Life Scale (24).

The List of Personal Values was divided into two parts: one containing nine symbols of happiness; and the other presenting 10 personal values. From the presented nine symbols of happiness, the respondents were to choose five and assign numbers from 5–representing the most important to 1–for the least important. The second group of 10 values was described analogically. The reliability of the tool, verified by the test-retest method, was 0.78 and 0.76 of the Personal Values List at an interval of 2 weeks, and after 6 weeks it was 0.72 and 0.62, which presents a satisfactory stability of the method (22).

The Satisfaction With Life Scale consists of five statements in which the respondents assessed the extent to which each of them relates to their life. Responses were given using a 7—point Likert scale, where: 7—I strongly agree, 6—I agree, 5—I tend to agree, 4—I neither agree nor disagree, 3—I tend to disagree, 2—I disagree, 1—I strongly disagree.

The results were measured and presented as a general indicator of the feeling of satisfaction with life ranging from 5 to 35 points (where 20 is considered neutral). Cronbach's alpha was used to measure the internal consistency which was 0.86. The test-retest method determined the stability of the results at a satisfactory level (0.85–0.93 at 3 week intervals; 0.87–0.88 at 6 week intervals; and 0.86 at 9 week intervals) (23).

### Outcomes

The primary outcomes described the percentage of people who donated blood at least once in their life. The secondary outcomes included sociodemographic factors, personal values and life satisfaction.

### Statistical methods

Intergroup differences (blood donor vs. non- donor) for categorical data were assessed using the Chi-square test. Mann-Whitney test was used for continuous data–normality of data distribution (age, life satisfaction) was tested with Shapiro-Wilk test. Categorical data were shown as absolute numbers and percentages, while continuous data were presented as medians, upper and lower quartiles.

A multiple logistic regression was carried out to assess the relationship between dependent variable “being a blood donor” (yes/no) and independent variables such as: age; gender; marital states; having children; having siblings; education level; employment status; place of residence; religious self- identity; belief that blood donation is safe; and life satisfaction. First, a simple logistic analysis was performed to select predictors–a variable which had a *p*-value < 0.1 was entered into the multiple regression model. Backward elimination technique was used to build an effective model. The Hosmer-Lemeshow test of the goodness of fit suggests the model is a good fit to the data as *p* > 0.05. Nagelkerke's *R*^2^ describes the proportion of variance in the outcome that the model successfully explains. The Wald statistics were used to test the significance of individual coefficients in the model. The odds ratio with 95% confidence interval was also calculated.

All calculations were performed using STATISTICA v.13.3. [TIBCO Software Inc. (2017), Krakow, Poland]. For all statistical analyses, values of *p* < 0.05 were considered significant.

## Results

### Characteristics of the respondents

The detailed characteristics of the study group (*n* = 770) are presented in Table 1. Thirty four percentage of the surveyed group were people who declared that they had donated blood at least once in their lives. The median age in this group was 37 years (mean 38.6 ± 12.9) and did not differ significantly (*p* > 0.05) from the median age in the group of non-donors (median 36 years; mean 37.9 ± 13.03). In both groups, a similar level of education was also noted–secondary education was dominant (50.9% of all respondents). Blood donors, like non-donors, were mostly urban residents (82.4 vs. 78.7%), Catholics (68.3 vs. 65.0%), and had siblings (84.3 vs. 79.3%). It should be emphasized that people who donated blood–compared to people who never donated blood–were more often married (69.1 vs. 42.9%; *p* = 0.001) and had children (59.5 vs. 48.6%; *p* = 0.004), and had a regular source of income in the form of paid work or disability/retirement benefits (71.8 vs. 61.4%, *p* = 0.004). There were also differences between the groups regarding gender–there was a higher proportion of men in the donor group than in the non-donor group (58.0 vs. 41.1%; *p* = 0.00001).

**Table 1:** Sociodemographic profile of blood donor and those who have never given blood.

**Variables**	**Ever given blood**		***Statistics values***	***P*-value**
	**Yes (*****n*** = **262)**	**No (*****n*** = **508)**		
Age (years)	37 [28; 48]	36 [27; 47]	Z = −0.72	0.470
**Gender**				
Male	152 (58.0)	209 (41.1)	*χ^2^ = * 19.76	0.00001
Female	110 (42.0)	299 (58.9)		
**Married**				
Yes	181 (69.1)	218 (42.9)	*χ^2^ = *10.47	0.001
No	81 (30.9)	290 (57.1)		
**Children**				
Yes	156 (59.5)	247 (48.6)	*χ*^2^ = 8.26	0.004
No	106 (40.5)	261 (51.4)		
**Siblings**				
Yes	221 (84.3)	403 (79.3)	*χ*^2^ = 2.83	0.092
No	41 (15.7)	105 (20.7)		
**Education level**				
University	96 (36.6)	156 (37.0)	*χ*^2^ = 4.75	0.190
High school	132 (50.4)	260 (51.2)		
Vocational education	23 (8.8)	65 (12.8)		
Primary school	11 (4.2)	27 (5.3)		
**Permanent source of income**				
Yes	188 (71.8)	312 (61.4)	*χ*^2^ = 8.11	0.004
No	74 (28.2)	196 (38.6)		
**Place of residence**				
City	216 (82.4)	400 (78.7)	*χ*^2^ = 1.48	0.224
Village	46 (17.6)	108 (21.3)		
**Religious self-identity**				
Catholic	179 (68.3)	330 (65.0)	*χ*^2^ = 0.87	0.351
Not religious	83 (31.7)	178 (35.0)		

Age were presented as median, upper and lower quartile. Categorical variables were presented as absolute numbers and percentages.

#### The practice of blood donation and perceived blood transfusion safety

In the group of respondents previously declaring blood donation (*n* = 262, 100%), 36.6% of the people donated blood once (*n* = 96), while 46.2% had donated blood more than once (*n* = 121). Honorary Blood Donors were only men (*n* = 45; 17.2%). The group of non-donors constituted 66% of all the respondents (*n* = 508). 20.9% of people from this group declared that they never intended to donate blood (*n* = 106), while the rest had health contraindications (*n* = 246; 48.4%) or considered donating blood in the future (*n* = 156, 30.7%). Respondents who donated blood (*n* = 10; 1.3% vs. *n* = 51; 6.6%; χ*2* = 9.17; *p* = 0.002) and with higher education (*n* = 12; 1.6 vs. 49; 6.7%; 4.50; *p* = 0.03), were less likely to consider this procedure dangerous than those who had never been a donor or had lower education (i.e., high school, vocational education, primary school).

#### Blood donation and the list of personal values

**Figure 2:**
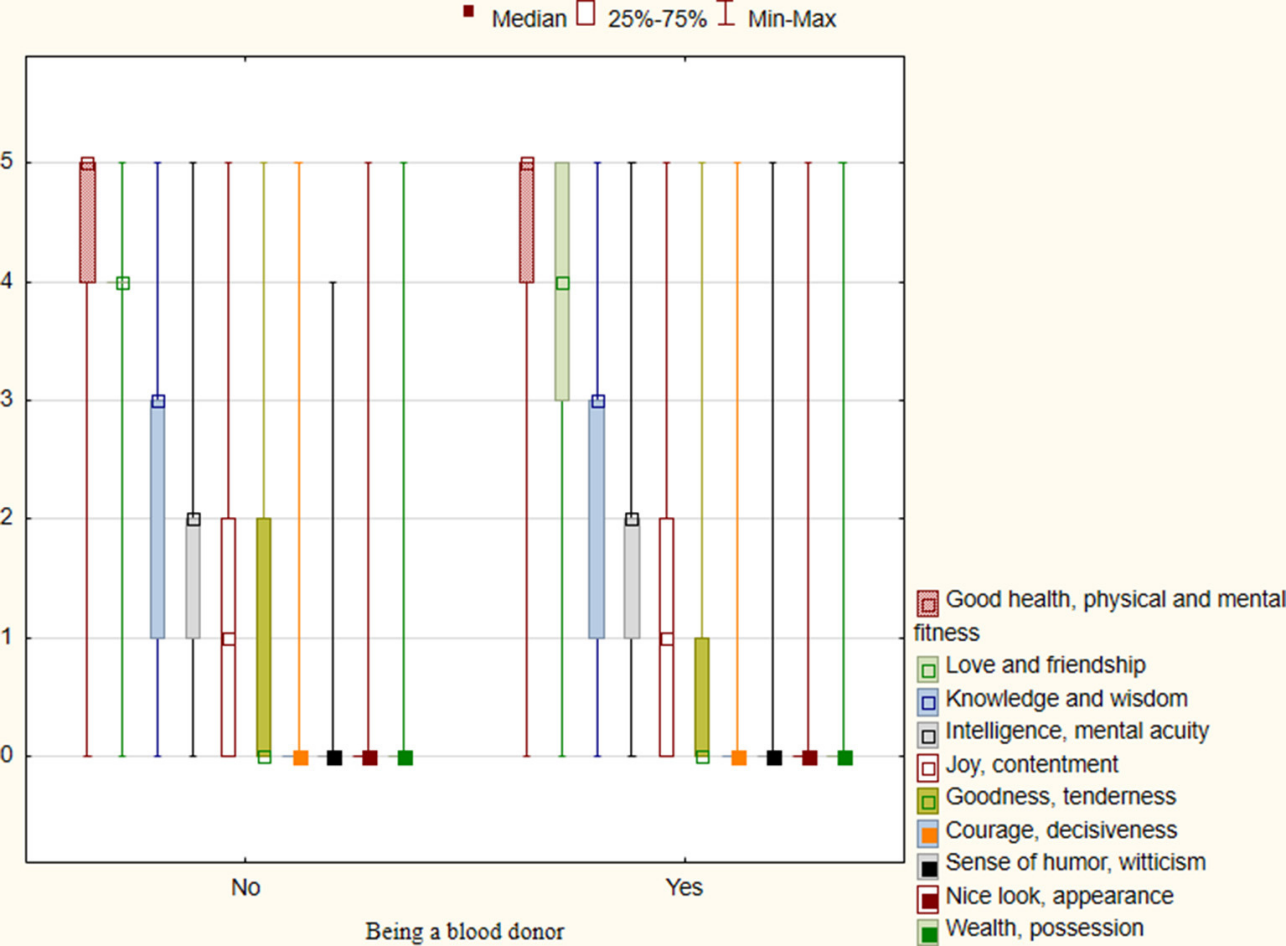
Categories of personal values for respondents who are not (*n* = 508) and who are blood donors (*n* = 262).

**Figure 3:**
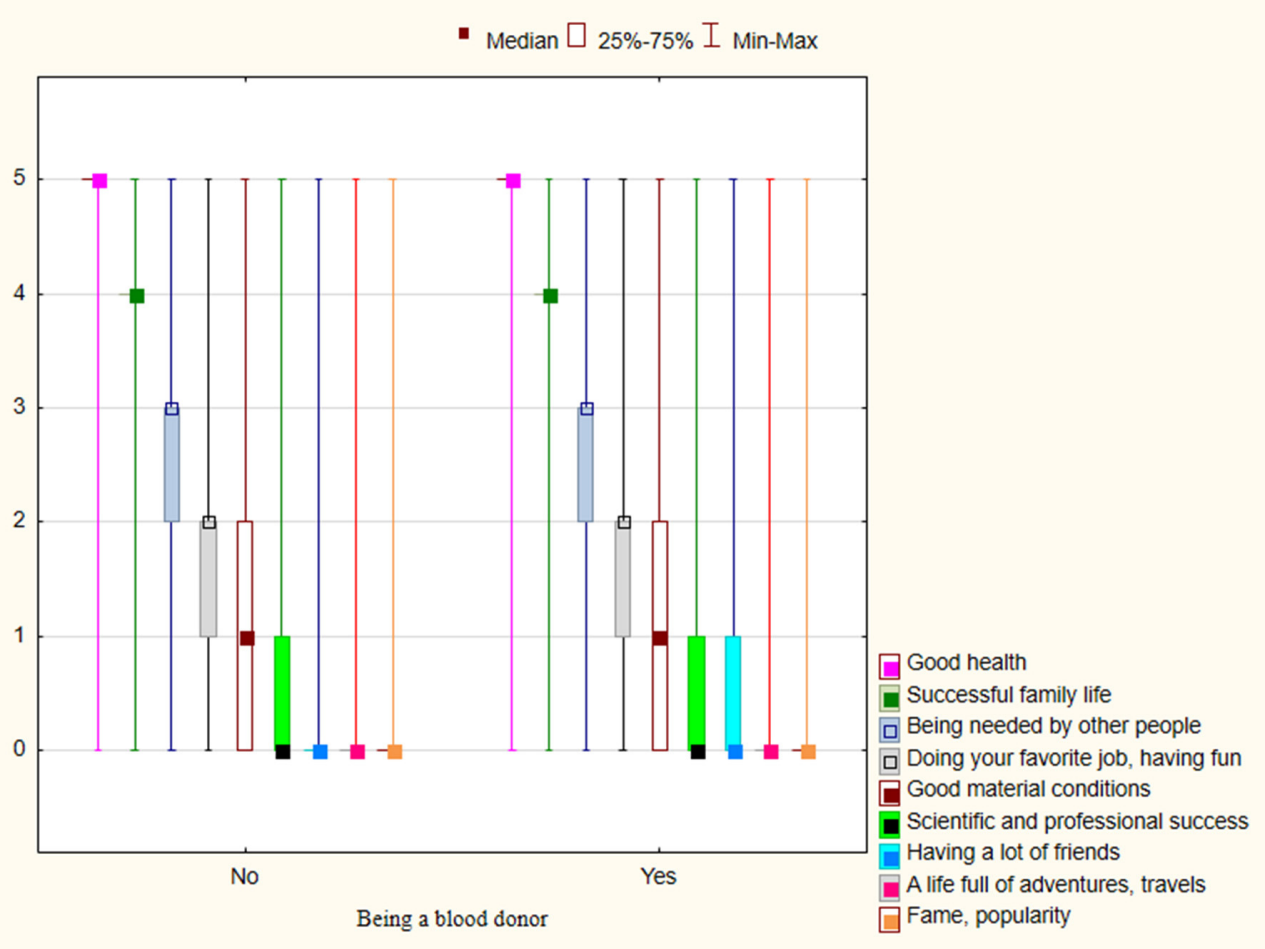
Distribution of symbols of happiness for respondents who are not (*n* = 508) and who are blood donors (*n* = 262).

Analyzing the results presented in Figures 2, 3, we noticed that the same categories of personal values and symbols of happiness were important for both the group of blood donors and non-donors. The five most important personal values for respondents were: good health; physical and mental fitness; love and friendship; knowledge and wisdom; intelligence; mental acuity, and joy and contentment. The five most frequently chosen symbols of happiness were: good health; successful family life; being needed by other people; doing your favorite job, having fun; and good material conditions. It is worth noting that people who declared that they would never give blood chose personal values and symbols of happiness in the same order as presented above.

### Blood donation and life satisfaction

The median total scores of The Satisfaction with Life Scale of blood donors was significantly higher than those of non-donors: 20 (15; 26) vs. 19 (15; 23); Z = −1.99; *p* = 0.046. When analyzing in detail the variables assessed by the respondents in the Satisfaction with Life Scale, it should be emphasized that statistically significant intergroup differences (*p* < 0.05) concerned only two statements: “In most aspects, my life is close to my ideal,” “If I could live my life again, I wouldn't change almost anything.” The median responses to the other three statements, i.e., “My living conditions are excellent,” “I am satisfied with my life,” “So far I have achieved the important goals I want in my life,” were not significantly different (*p* > 0.05).

### Factors determining being a blood donor

Factors determining being a blood donor are presented in Table 2. Male respondents (OR = 1.47; Cl95%: 1.26 to 1.72), and those who believed that blood donation is safe (OR = 1.71; Cl95%: 1.20 to 2.43), and who were married (OR = 1.31; Cl95%: 1.11 to 1.54) would more likely donate blood. Education level correlated negatively with the being a blood donor (OR = 0.81; Cl95%: 0.66 to 0.99).

**Table 2:** Factors determining being a blood donor.

**Variables**	***B***	***SE (B)***	***Wald test***	***p***	***OR (Cl 95%)***
**Simple logistic regression**					
Blood donation is safe	0.52	0.17	8.50	0.003	1.68 (1.18–2.37)
Male	0.26	0.08	10.38	0.001	1.30 (1.11–1.52)
Married	0.23	0.07	9.61	0.002	1.26 (1.09–1.46)
Having children	0.22	0.08	8.22	0.004	1.25 (1.07–1.45)
Having siblings	0.17	0.10	2.82	0.09	1.18 (0.97–1.44)
Education level	−0.20	0.10	4.07	0.044	0.82 (0.67–0.99)
Having permanent source of income	0.23	0.08	8.05	0.004	1.26 (1.07–1.48)
Life satisfaction	0.02	0.01	3.70	0.054	1.02 (1.00–1.05)
**Multiple logistic regression model**					
Blood donation is safe	0.53	0.18	8.75	0.003	1.71 (1.20–2.43)
Male	0.39	0.08	24.0	0.000	1.47 (1.26–1.72)
Married	0.27	0.08	10.49	0.001	1.31 (1.11–1.54)
Education level	−0.21	0.10	4.08	0.043	0.81 (0.66–0.99)

B, regression coefficient; SE, Standard error; OR, Odds ratio; CI, Confidence interval; Multivariable logistic regression model: R^2^Nagelkerka, 0.083; Hosmer-Lemeshow, 7.217; *p* = 0.205.

## Discussion

The results of the study revealed that more than one-third of the participants had a history of blood donation. Perceived blood donation safety and sociodemographic factors such as being a male and being married as well as having completed high school may be the determinants of more likely being a blood donor.

Our study showed that 34% of respondents aged 18–65 have donated blood at least once in their lives. This percentage is higher than the data from the study conducted in 2014 among citizens of 28 European Union countries aged 15 and over (*n* = 27,868). In the aforementioned study, 27% of Polish participants had a history of blood donation, while at the same time the percentage of German and French participants who donated blood was 38 and 52%, respectively (25). Also, every fifth respondent from the group of non-donors declared that they never intend to donate blood in the future, despite the lack of health contraindications. Perhaps one of the reason for this negative attitude was blood donation fear during the COVID-19 pandemic. Tripathi et al. (26) reported that the fear of infection with the SARS-CoV-2 virus was the most important factor deterring people from donating blood.

The results of our study show that people who view blood donation as a safe procedure are more likely to donate blood. According to a survey by Makowicz et al. (27) (*n* = 2,387), the vast majority of Polish voluntary blood donor respondents are convinced of no side effects of frequent blood donation–they rarely believed in the myths that regular blood donation is addictive, causes overproduction of red blood cells, leads to high blood pressure/hypertension, or that the donor can become infected during blood donation. Moreover, the willingness to donate blood in the future is associated with positive beliefs about blood transfusion safety (25), which are determined by higher average education, life expectancy and Gross Domestic Product (28).

In our study, gender disparity in blood donation has been reported–males consisted of 58% of blood donors. According to Kryczka et al. (29) almost four times more Polish men than women donated blood in 2020 and 2021 in the Military Blood Donation. Greater proportion of male blood donors were also found in German (54.5%) (30), Spanish (52.3%) (31), and Italian (50.3%) (12) populations. Most Turkish donors who participated in Ulukanlig et al. study (32) were also male. Only 5% of regular donors, 9% of returning donors and 23% of first-time donors were female. Female dominance over male blood donors (63.1 vs. 36.9%) was found among undergraduate students (full-time and part-time) from a university in Hong Kong (13). Suemnig et al. (33) noticed that the gender of the donor was associated with the number of blood donations over the period of 12 months−60.3% of women donated blood only once or twice, while 61.0% of men donated three or more times. Ulukanligi et al. (32) confirmed that women are still under-represented among blood donors and donate blood less regularly than men. Gender differences in donation patterns can be partly explained by their absence due to pregnancy and lactation (34). Additionally, women are also more prone to iron depletion than men (35) because of menstrual blood loss, which results in low hemoglobin and donation deferrals. The results of Clement et al. (36) study suggested that temporary deferrals hurt future donation behavior.

In this study, blood donation practices are tied to marital status–those participants who were married were more likely to be blood donors. The result is in agreement with studies conducted in Canada (37) and Greece (38). In Brazil, people who were divorced or widowed were associated with a self-defined inability to donate blood (39). In contrast, another study from Ethiopia found no relationship between marital status and donating (40). In this matter, studies regarding donor marital status showed mixed results.

In terms of education, we found that people who completed high school were more likely to donate than those who graduated university, similar to Guglielmetti Mugion et al. (12) study among Italian citizens. Suemnig et al. (33) found that in the German population donation status and frequency were correlated with the educational status of the donor–donors with higher-grade education (>10 years of school) comprised the biggest group, whereas the group of frequent donors was dominated by those with 10 years of school education. In a Spanish study conducted by Romero-Domínguez et al. (41) a higher frequency of donations was observed among individuals with a university education than lower educational, however, those highly educated were the least motivated to continue to donate and deepen their commitment to the cause.

In our study, donations were motivated neither by life satisfaction nor personal values. The same personal values (good health, physical and mental fitness; love and friendship) and symbols of happiness (good health; successful family life; being needed by other people) were important to both donors and non-donors. However, research confirms that altruism is the most common motivational factor for blood donation (33). Tey et al. (42) found that “helping people who need blood transfusion” was the central motivation for deciding on blood donation. That motivations directly links to such values as: “happy;” “moral duty;” “humanitarianism;” and “social responsibility.”

## Strengths and limitations

A strength of this study is the use of validated questionnaires to assess personal values and satisfaction with life. The weakness of this study is using secondary datasets–the available data may not contain the exact variables that best fit the purpose of this study. Among the factors that could have distorted the answers provided by the respondents was conducting the survey only among the Polish citizens.

## Conclusion

The results of the study revealed that more than one-third of the participants had a history of blood donation. The Polish donor is most likely a married, middle-aged man with a secondary education, and who perceives blood donation as safe.

## Implications for clinical practice

Knowing the profile of blood donors is important for formulating and monitoring strategies for recruiting and retaining active blood donors. The profile of donors may change over time (30) and may be conditioned by the demographic, socio-political situation or blood management programs of a given country. Therefore, it is crucial to provide updated knowledge about demographic profile and motivators (e.g., personal values) of both blood donors and non-donors.

Based on the gathered data, it can be concluded that the group most involved in blood donation in the Polish population are married men, with an middle school education, who consider blood donation as a safe procedure. The indicated factors affecting the readiness to donate blood may be a guide to profiling campaigns and communication channels (social media) with both current and potential future donors. Expanding the blood donor base also requires targeting recruitment campaigns toward women and university graduates along with promoting the safety of blood donation.

## Data availability statement

The original contributions presented in the study are included in the article/supplementary material, further inquiries can be directed to the corresponding author.

## Author contributions

Conceptualization, supervision, project administration, resources, and data curation: PK. Methodology and software: PK and LT. Formal analysis: LT. Writing—original draft preparation: BS, LT, PK, and ET. Writing—review and editing: BS, LT, PK, ET, and WM-D. Visualization: LT and WM-D. All authors have read and agreed to the published version of the manuscript.
